# Non-Cutaneous Melanoma, Findings and Prognostic Value of FDG PET/CT: A Case Series of 23 patients and review of the literature

**DOI:** 10.22038/AOJNMB.2022.61517.1433

**Published:** 2022

**Authors:** Bahare Saidi, Babak Fallahi, Armaghan Fard-Esfahani, Alireza Emami-Ardekani, Mohammad Eftekhari

**Affiliations:** Research Center for Nuclear Medicine, Tehran University of Medical Sciences, Tehran, Iran

**Keywords:** Non-cutaneous melanoma, PET/CT, Metastases, Prognosis

## Abstract

**Objective(s):**

Non-cutaneous malignant melanomas (NCM) are rare malignancies. Due to their nonspecific symptoms, they present later in life. The value of FDG PET/CT in this group of patients is not clear. The aim of this study is to assess the role of FDG PET/CT in the management of NCM and its prognostic implication.

**Methods:**

We retrospectively selected twenty-three patients with a diagnosis of NCM evaluated with FDG PET/CT in Shariati hospital between 2019 and 2021. The PET/CT data were reviewed and compared with available conventional imaging findings. Five patients died within five months. The surviving patients were followed within a time interval of 7 to 27.5 months after their PET/CT study, regarding their disease status.

**Results:**

Among 23 patients (8 ocular, 5 sinonasal, 3 pharyngeal, 2 anorectal, 2 vulvovaginal, and 3 unknown primaries), PET/CT was able to detect residual primary disease, assess treatment response, and reveal or exclude metastases. Additional lesions compared to conventional imaging were found in five, while in one with brain metastases PET/CT was unable to detect lesions on MRI. Thirteen patients had negative PET/CT finding of which 11 (85%) did not have remarkable finding on follow-up. Metastatic disease was recognized in eight. Patients with extensive metastases on FDG PET/CT had a poorer outcome.

**Conclusion:**

Similar to cutaneous melanoma, PET/CT is valuable in the management of NCM patients and is superior to conventional imaging modalities, with the exception of brain metastases. Patients with negative PET/CT findings have a better outcome as opposed to patients with significant positive PET/CT findings.

## Introduction

 Noncutaneous melanomas (NCM) are very rare tumors, arising from different muco-cutaneous areas in the head and neck region (ocular, sinonasal and pharynx), as well as anorectal and genitourethral areas (1, 2). Other very rare sites of tumor origin such as adrenal, esophagus and biliary tract have also been reported (1). The time course and prognosis of these tumors are different from the cutaneous malignant melanomas (1), as they usually have an indolent course, and are detected later in life 

because of their nonspecific symptoms (i.e sinonasal melanomas are known to occur in the elderly, presenting with epistaxis, being very aggressive and representing a worse prognosis) (3,4).

 Conventionally CT and MRI were the imaging modalities of choice for evaluating melanoma lesions. Due to their vascularity, the lesions appear with the radiocontrast enhancement of CT images. Also, they are T1 hyper intense, T2 hypointense and have restriction on diffusion weighted images on MRI (1). 

 The classic T1 hyperintensity is present in only 24-47% of melanocytic lesions which correlates with the amount of melanin in these lesions (1, 5). These lesions are typically FDG avid, with false negative results, more commonly reported in nodal lesions less than 5 mm (6, 7). FDG PET/CT is gaining interest in staging and follow-up of NCM patients. NCCN guidelines 2021 recommend FDG PET/CT, after biopsy confirmation of mucosal malignant melanoma of head and neck region, to rule out metastatic involvement (8). In this study, we present the PET/CT findings of different types of NCM referred to our center for different indications. We also try to elucidate the additional value of the PET/CT as compared to the other imaging modalities.

## Methods

 We retrospectively reviewed all PET/CT studies performed at our center between2019-2021 and selected the NCM patients. A total of 23 patients were included, 19 were pathologically confirmed, and remaining four were diagnosed as ocular melanomas by imaging and clinical evaluation. The patients were categorized according to the site of tumoral lesion into six groups, ocular, sinonasal, pharyngeal, anorectal, female genital tract and finally unknown primary. The patients were primarily referred to us for staging, evaluation of metastases, surveillance or monitoring of treatment response. PET/CT scans were reviewed for the detection of primary site, local recurrence and distant metastases. The data on available conventional images were correlated with PET/CT findings. Subsequently, the patients were followed at the end of the study, regarding their disease status.

***FDG PET-CT Imaging***

 Total Body imaging was performed approximately 60 minutes after intravenous (IV) administration of 0.14 mCi/kg FDG. The images were obtained using Siemens Biograph 6, TruePoint PET-CT scanner with first craniocaudal CT (110 kV, 80 mAs and pitch index of 1.3) followed by caudocranial PET imaging in 3D mode and 3-4 min/bed position duration. The reconstruction was based on ordered subsets expectation maximization (OSEM) algorithm (2 iterations; 21 subsets) and Gaussian filter were utilized post-reconstruction to smooth the images (4.0 mm FWHM). Syngo software TrueD (Siemens) was employed to estimate the SUVmax of the primary and metastatic lesions.

***FDG PET/CT Evaluation***

 The PET/CTs were reported by experienced nuclear physicians. The PET/CTs had been evaluated for the presence of focal abnormal FDG uptake on attenuation-corrected images as well as non-attenuation corrected images (to evaluate any possible skin lesions). Standard uptake values (SUV) were obtained for all primary and metastatic lesions.

## Results

 A total of 23 patients were evaluated. The patients’ characteristics are summarized in table 1.

**Table 1:** Patients’ characteristics

	**Type**	**Age**	**Gender**	**Primary lesion**	**Pathology report**
1	Ocular	48	M	Ocular	NA
2	Ocular	28	M	Choroid	Diameter: 17 mm Thickness: 8 mm (pT3)
3	Ocular	52	F	Choroid	Large size Sclera involved Vortex Vein involved
4	Ocular	37	M	Ocular	NA
5	Ocular	38	M	Choroid	2.5 cm x 1 cm invades fibroadipose tissue, lymphoid tissue involved
6	Ocular	51	M	Choroid	Spindle B cell type, tumor around optic disc Focally involved inner layer of sclera Medium size
7	Ocular	53	F	Ocular	NA
8	Ocular	58	F	Ocular	NA
9	Sinonasal	67	M	Nasal (Figure 1A)	IHC positive for malignant melanoma
10	Sinonasal	40	M	Sinonasal	NDA
11	Sinonasal	87	M	Nasal	Perineural invasion
12	Sinonasal	83	F	Nasal	Lymphovascular invasion IHC: malignant melanoma
13	Sinonasal	51	M	Nasal	5 cm
14	Pharynx	60	M	Hard palate	Unifocal, 3 cm Bony tissue involved
15	Pharynx	40	M	Right tonsil (Figure 1C)	Tonsil extensively involved by malignant melanoma
16	Pharynx	30	M	Nasopharynx	Nodular type Breslow thickness: 12 mm Clark at least IV Margins involved
17	Anorectal	74	F	Anorectal	NDA
18	Anorectal	66	F	Perianal (Figure 1E)	2.8 cm x 2 cm thickness: 1.5 cm lymphovascular invasion Close to margin
19	Vulvovaginal	58	F	Vulve	Lentigo maligna Breslow: 1 mm Clark III Margins involved
20	Vulvovaginal	74	F	Vulve (Figure 1D, G)	Clark IV, Breslow 7 mm Ulceration Vascular invasion
21	Unknown Primary	54	M	Unknown (Figue 1B)	Lymphoid node tissue of left parotid Extranodal extension
22	Unknown Primary	32	F	Unknown (Figure 1F)	Left inguinal node, metastatic malignant melanoma
23	Unknown Primary	60	F	Unknown	Liver: metastatic malignant melanoma Periaortic lymph node: metastatic malignant melanoma

NA: not applicable; NDA: no data available

 The median age was 53 years (range, 28-87 years). Ocular melanoma was the most common NCM (8/23 patients). Imaging findings are summarized in Table 2.

 Among 13 patients with negative PET/CT, 11 (85%) patients did not have any remarkable finding during follow-up period of 7-28 months. None of ocular NCM revealed any metastasis on FDG PET/CT images. 

**Table 2:** Details of imaging findings and follow-ups in each patient

	**Primary site**	**Primary site resected, and treatment**	**Indication**	**Conventional Imaging findings or ** **previous PET/CT data**	**FDG PET/CT finding**		**Finding and Treatment after PET/CT**	**Follow-up**
					**Primary site**	**Hyper metabolic Metastases**		
1	Ocular	No brachytherapy	Surveillance	CT: Atypical hemangioma of liver Otherwise, NRF	Negative	Negative	No treatment	Sonography: NRF
2	Ocular	Yes enucleation	Evaluation of Recurrence	MRI: lesion with enhancement, post-septal region CT: Anterior wedge deformity of T9 with irregularity of end-plates. otherwise NRF	Negative	Negative Degenerative changes T9-T10	No treatment	Orbit-CT: negative
3	Ocular	Yes enucleation	Surveillance	Brain MRI: negative CXR: negative	Negative	Negative	No treatment	NRF
4	Ocular	No Brachytherapy	Surveillance	NDA	Negative	Negative	No treatment	Enucleation: Necrotic lesion peripheral pigmentation. MM could not be ruled out
5	Ocular	Yes enucleation	Surveillance	PET/CT: negative	Negative	negative	No treatment	MRI: Suspicious orbital lesion, biopsy negative
6	Ocular	Yes enucleation	Surveillance	Chest CT: suggestive of COVID-19 Otherwise NRF		Negative	No treatment	NRF
7	Ocular	No Brachytherapy	Surveillance	NDA	Negative	Negative	No treatment	NRF
8	Ocular	No brachytherapy	Surveillance	Abdominal sonography: negative Chest-CT negative	Negative	Negative Multinodular goiter	No treatment	NRF
9	Sinonasal	No	Staging	MRI: Enhancing mass right nasal cavity, extension to adjacent sinuses. Abdominal sonography: heterogeneous right adrenal gland	Hyper metabolic mass in right nasal cavity, extension to adjacent sinuses.	- Left ethmoidal lesion. - Bilateral huge adrenal metastases. - Mediastinal lymph nodes - Gastric lesion	Advanced Stage No treatment	Died (two weeks after PET/CT)
10	Sinonasal	Yes	Evaluation of metastasis	NDA	Negative	- Widespread bone - Subdermal lesion, lumbar region - Lymph node (precaval)	No Treatment Advanced stage	Died (one month after PET/CT) Advanced stage melanoma and scleroderma
11	Sinonasal	Yes, Radiotherapy, chemotherapy	Evaluation of Recurrence	NDA	Negative	- Right maxillary sinus - Left nasal cavity - Lung - Bone	Radiotherapy Chemotherapy	Under treatment
12	Sinonasal	No	Staging	CT: 30 mm x 18 mm x 20 mm right nasal cavity Otherwise: NRF	Negative	Negative	No treatment	NRF
13	Sinonasal	Yes	Surveillance	PET/CT: negative	Negative	Negative	No treatment	NRF
14	Pharynx	Yes	Evaluation of Metastasis	NDA	Negative	Negative	Radiotherapy	NRF
15	Pharynx	Yes Radiotherapy chemotherapy	Evaluation of Metastasis	CT: (before tonsillectomy) right tonsil mild heterogeneous enhancement, extension to right lateral part of soft-palate Lymph nodes level II	Possible residual disease in bed of right tonsillectomy	- Right level II metastatic cervical lymphadenopathy	Radiotherapy	Coma, radiotherapy complications
16	Pharynx	Yes radiotherapy	Surveillance	Post-treatment PET/CT: Negative	Negative	Negative	No treatment	PET/CT: negative NRF
17	Anorectal	Radiotherapy	Evaluation of Response to treatment	MRI: anorectal lesion, L2 hemangioma, L1 Compression fracture Lymph node in mediastinum axillary	Anorectal wall thickening with mild metabolic activity and adjacent lymph nodes	- Deposits/ lymph nodes in abdomen - Lymph nodes in: cervical mediastinum right axillary - Deposits in: subcutaneous intramuscular region in left arm, thorax right posterior abdominal wall - Bilateral adrenals, - L2 - Gastric thickening	No treatment (advanced stage)	Died (three months after PET/CT)
18	Anorectal	No	Staging	NDA	Metabolically active lesion in anal canal	Negative	Surgical resection	Follow-up PET/CT: negative
19	Vulvovaginal	Yes	Surveillance	CT: Lytic in the body of C5 Otherwise NRF	Negative	Negative degenerative changes in spine, especially L5	No treatment	Follow-up PET/CT: negative NRF
20	Vulvovaginal	Yes Brachytherapy Interferon	Evaluation of Recurrence	Sonography: multiple lesions in the liver, heterogeneous lesion in left adnexa	Tumoral mass on the left side of vaginal cuff	- Lymph nodes in abdominopelvic, - Bone - Liver - Spleen - Lung - Adrenal	Advanced Stage, No treatment	Died (three days after PET/CT study, advanced disease)
21	Unknown Primary	No	Staging	Cervical CT: Heterogeneous lesion in the left parotid, resected	Negative	Right parotid lesion.	FNA right parotid lesion: MM MRI: cerebral metastases. Chemotherapy	Died (5 months after PET/CT)
22	Unknown Primary	No, Radiotherapy	Evaluation of Response to Treatment	Staging PET/CT: multiple hypermetabolic lytic metastases	Negative	Multiple sclerotic lesions without metabolic activity (healed lesions)	No treatment	PET/CT: stable
23	Unknown Primary	No	Staging	MRI: lesions in liver segments V and VI Lymph nodes, portohepatis subdiaphragmatic EUS: lesions both liver lobes Lymph nodes: periportal, celiac, periaortic,	Negative	- Large lesion in right liver lobe. - Lymph nodes: right retrocrural, portahepatis, celiac portocaval. - Foci of metabolic activity in T12, L1, L3 and proximal right femur	chemotherapy	Not favorable status

MM: malignant melanoma; NRF: no remarkable finding; NDA: no data available

**Figure 1:**
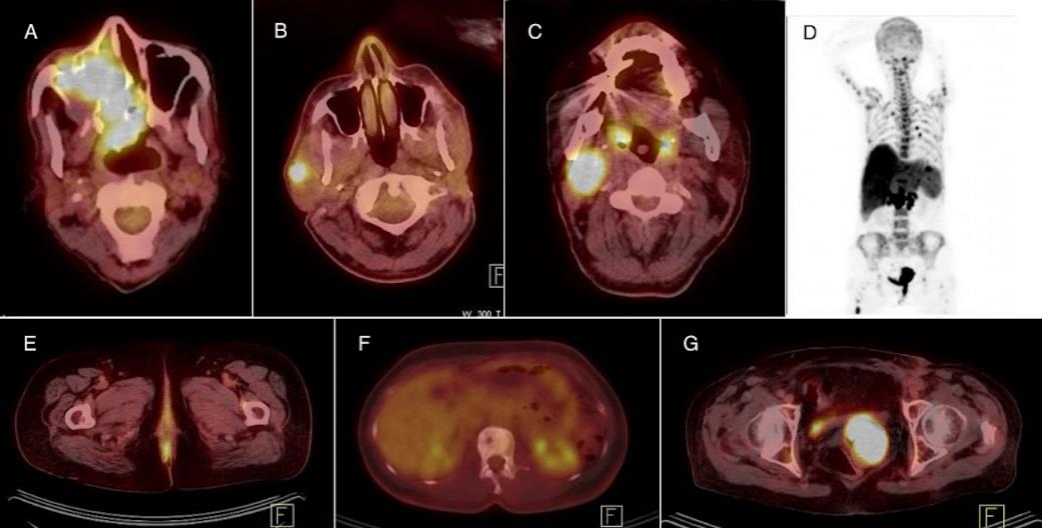
(**A**) FDG PET/CT of an 87-year-old male with noncutaneous melanoma (NCM) in the right nasal cavity with extension to adjacent sinuses (case 9). (**B**) FDG PET/CT of a 54-year-old male with NCM, presenting after resection of left parotid mass. (Additional metastatic lesion is noted in the right parotid, not recognized on patient’s CT scan, case 21). (**C**) A 40-year-old male with NCM of right tonsil. (FDG PET/CT reveals residual disease at the site of right tonsillectomy as well as metastatic lymph nodes in right cervical zone II, case15). (**D**) MIP image of a 74-year-old female with vulvar NCM revealing extensive metastases, case 20. (**E**) FDG PET/CT of a 66-year-old female with NCM in the anal canal (case 18). (**F**) FDG PET/CT image of a 32-year-old female with NCM of unknown primary currently revealing metabolically inactive sclerotic bone lesions in the site of previously lytic bone metastases, indicative of complete response to treatment (case 22). (**G**) FDG PET/CT image of the patient in MIP image (**D**), revealing local recurrence (case 20)

 Metastatic disease was recognized in eight patients (8/24), (including three sinonasal, one pharyngeal, one anorectal, one vulvovaginal and two unknown primary). Among three patients being evaluated prior to lesion resection, PET/CT revealed hyper metabolic primary tumor in two (case 9 and 18) (Figure 1, A, E) and no metabolic activity was noted at the primary site in the third (case 12). Among 4 patients referred after lesion resection, possible residual disease and metastases were noted in one (case 15) (Figure 1, C) while metastatic lesions were noted in two. Of two patients evaluated for the treatment response, complete metabolic response was noted in one (case 22) (Figure 1, F) and progressive disease was noted in the other (case 17). Extensive metastases were noted in five patients (cases 9, 10, 11, 17, 20, 23) (Figure 1, D) of whom four cases died during an interval of less than four months. PET/CT in three cases of indeterminate lesion on conventional imaging (23% of patients with available current imaging data), was able to characterize the benign nature of the lesions, including an atypical liver hemangioma which showed no increased metabolic activity, and was stable on follow-up (case 1). Two suspicious bone lesions were sites of degenerative changes demonstrating no metabolic activity and were also characterized as benign (case 2 and 19). Additional lesions were detected on PET/CT in comparison with conventional imaging in 38% (5/13) (one sinonsal, one anorectal, one vulvovaginal and two unknown primary) (Figure 1B). In one case MRI detected brain metastases which were not recognized on PET/CT (case 21).

 On follow-up, five (5/24) patients died as a consequence of advanced stage of disease, four of these patients had widespread metastases and one with cerebral and parotid metastases. Two patients continue to have unfavourable clinical status, one of the patients currently in coma following complications of radiotherapy treatment and the other has multiple metastases. Among 13 patients with negative PET/CT, 11 (84%) patients did not have any remarkable finding during follow-up period (7-27.5 months). 

## Discussion

 NCMs are rare malignant diseases. They commonly arise from ocular as well as different mucosal regions (2). There is also a group of metastatic melanomas with no distinct origin (2, 9). NCMs are more aggressive than cutaneous melanoma with a reported 5-year overall survival of 25% (10). The value of FDG PET/CT in diagnosis, follow up and response assessment is not yet fully recognized (2).

***Ocular NCM***

 The value of FDG PET/CT in the detection of primary eye melanoma remains debatable. Although it is more likely to detect primary lesion by FDG PET/CT in nodular rather than diffuse type of ocular melanoma (11), there has been reports of low uptake even in nodular type (12). As a result, the main indication of PET/CT in these cases seems to be the detection of distant metastases. Of the eight patients with ocular melanomas in our series, none demonstrated any evidence of metastases on surveillance studies. In a series of 52 patients referred for initial staging two patients revealed distant metastases on PET/CT studies (liver, lymph node, brain and bone), both were considered to have large primary ocular NCMs (13). Although size and extension are important when referring patients for PET/CT evaluation, even with large lesion and lymphovascular invasion, no metastatic disease was recognized in our ocular group of patients (case 3). Liver metastases from ocular melanoma on FDG PET/CT have result in conflicting findings (14-17). The SUV_max_ in liver metastases is lower in ocular NCM compared to cutaneous melanoma (15). In the study by Strobel et al. 16 liver metastases were negative on FDG PET/CT (15). 

 Other studies have reported good sensitivity and positive predictive value for liver metastases (16, 17). Although FDG PET/CT could be limited in the evaluation of liver metastasis, it might still be valuable in supporting a malignant vs. benign etiology of an equivocal liver lesion on conventional imaging (case 1).

***Sinonasal NCM***

Sinonasal NCMs are rare form of mucosal malignant melanoma. They usually present later in life with non-specific symptoms. Cervical lymphadenopathy is not common in these patients and metastases occur via hematogenous route. If detected early, resection is the mainstay of therapy. The efficacy of adjuvant radiotherapy is controversial in these patients, although frequently employed with high risk features (1). 

 In our series of patients, with nasal NCM, there was a lack of metabolic activity in the primary lesion in case 12 likely as a result of removal of the bulk of the lesion during biopsy. In case 10, FDG PET/CT was able to demonstrate the extent of primary lesion as well as metastatic involvement, including bilateral huge adrenal metastases. Haerle et al. evaluated 10 consective patients with sinonsal NCM with FDG PET/CT (18). In the patients presented for initial staging, similar to case 9, FDG PET/CT was able to detect the primary tumor and the results correlated to CT or MRI findings in the characterization of locoregional extension. Also, in their study FDG PET/CT was able to support the benign nature of equivocal CT findings which was confirmed on follow-up evaluation (18). In study by Agrawal et al., of 19 patients with head and neck NCM 11 were sinonasal NCM (19). In this study FDG PET/CT overlooked frontal lobe metastasis in a case of maxillary sinus NCM (19). Although PET/CT is valuable in detecting distant metastasis, it has limited value in the brain, where MRI is the imaging modality of choice (18, 19).

***Pharyngeal NCM***

 Pharygneal NCM is not a common malignancy, usually localized on palate and maxillary gingiva (20, 21). They appear as pigmented lesions and should be differentiated from benign and malignant lesions such as melanoplakia, blue nevi, physiologic pigmentation and poorly differentiated cancer (22). The use of radiotherapy as the primary treatment of oropharyngeal melanoma is controversial, and surgery is usually the main treatment option (21). Pharyngeal NCM usually presents in later stages and hence the prognosis is not favorable (21). Xiao et al. reported the high FDG avidity of a mass on the right wall of oropharynx which was subsequently characterized as malignant melanoma on histopathologic examination (23). In our series, FDG PET/CT was valuable in post-surgical evaluation of case 15, a case of advanced NCM of pharynx, demonstrating residual disease as well as involved lymph nodes. In the study by Agrawal et al. lymph node metastases were detected by FDG PET/CT in two patients with NCM of the palate (19). FDG PET/CT is valuable this group for detecting local disease as well as possible distant metastases.

***Anorectal NCM***

 Anorectal NCM is another rare mucosal melanoma. Diagnosis at early stage is difficult. They are usually misdiagnosed as other benign conditions such as hemorrhoids (24). These tumors are drained locally including in the inguinal and mesenteric lymph nodes and metastasize most commonly to the liver and lung (25). Local relapse is more common with the tumor volume ≥3.5 cm (26). Although, these lesions are FDG avid on FDG PET/CT, other conditions such as hemorrhoids, primary rectal adenocarcinoma and anal squamous cell cancer, can also show FDG avidity (27). MRI is considered superior for perirectal lymph node detection; however, PET/CT has higher detection rate for inguinal and pelvic lymph nodes (27). Due to rarity of anorectal NCM, the value of FDG PET/CT in these patients is less clear. In our series, FDG PET/CT was valuable in staging (case 18) and response assessment (case 17). In case 18, no metastatic disease was detected and the patient underwent wide local excision. This case did not show any recurrence or residual disease on the follow-up PET/CT performed 9 months later. Similar to cutaneous malignant melanoma, tumor thickness is an important prognostic factor in anorectal NCM and of importance when wide local excision or abdominopelvic resections are being considered of the extent of metastatic disease. In a case report by Li et al., FDG PET/CT revealed the full extent of metastatic lesions, including lymph nodes, lungs, liver and bone (28). Bulut et al., also reported the advantage FDG PET/CT in primary staging of anorectal NCM by demonstrating reactive lymph nodes with insignificant metabolic activity and no distant metastases prior to surgical resection (26). According to these studies FDG PET/CT is valuable in primary staging of anorectal NCM (4, 26-28). Moreover, as in case 17, it could be advantageous in the assessment of response to treatment.

***Vulvovaginal NCM***

 There have been rare reports of female vulvuvaginal melanoma evaluated by FDG PET/CT. Vishnoi et al. reported a case of malignant melanoma of the uterine cervix demonstrating metastases in inguinal lymph nodes on FDG PET/CT study (29). In cases of localized vaginal NCM, surgery is the main treatment option while chemotherapy mainly decarbazine and radiotherapy are appropriate for advanced or recurrence cases (31). The pathologic report of case 19 was lentigo maligna, the most common subtype of NCM located in female external genital region (32). In this case no evidence of metastasis or local recurrence was detected on PET/CT images, while in case 20, the patient with vulvar NCM presented with extensive local recurrence and distant metastases on PET/CT (Figure 1D, G), all were highly FDG avid. Tsai et al., reported rapid progression of case of NCM of uterine cervix. In this case FDG PET/CT also revealed extensive distant metastases in brain, breast, lung and lymph nodes (30). Thus, FDG PET/CT seems to be valuable in this group by demonstrating the presence and extent of metastases. The pathologic report of the primary resected lesion in case 20 showed high risk features, such as vascular invasion, ulceration and clark IV and a Breslow thickness of 7 mm. in cases with higher risk pathologic features, closer PET/CT follow-up with more caution over any metabolic activity above background, is recommended.

***Unknown Primary***

 In a subgroup of malignant melanoma, no site of primary origin can be detected. In a study by Egbert et al. BRAF mutation was recognized in 53% of a group of 44 patients of melanoma with unknown origin, which resembles that of cutaneous melanoma (9). The value of FDG PET/CT in this group has rarely been reported. In the study by murphy et al. similar to our three cases, FDG PET/CT did not identify the site of origin in a case of melanoma with an unknown origin (4). Although no site of origin could be detected in this group in our series, FDG PET/CT determined the metastatic lesions and was valuable in the assessment of treatment response.

## Conclusion

 These tumors are quite infrequent, and mostly have been reported as case reports or series. In our series with limited number of patients, FDG PET/CT was valuable in NCM patients for the assessment of residual tumoral lesion as well as for staging, assessment of recurrence, treatment response and determination of prognosis, while PET/CT was not suitable for the detection of primary lesion in the subgroup of unknown primary NCM. Patients with significantly positive PET/CT findings had poorer outcome, as opposed to most patients with negative PET/CT finding (85%), who had no remarkable finding on follow-up. In comparison to conventional imaging, PET/CT, being a whole body molecular imaging modality, seems superior in predicting outcome in NCM patients. MRI may be superior to FDG PET/CT for the diagnosis of cerebral metastases.
